# Serum Activin A as Brain Injury Biomarker in the First Three Days of Life. A Prospective Case—Control Longitudinal Study in Human Premature Neonates

**DOI:** 10.3390/brainsci11091243

**Published:** 2021-09-20

**Authors:** Dimitra Metallinou, Grigorios Karampas, Eleftheria Lazarou, Nikoletta Iacovidou, Panagiota Pervanidou, Katerina Lykeridou, George Mastorakos, Demetrios Rizos

**Affiliations:** 1 Department of Midwifery, University of West Attica, Ag. Spyridonos Street, 12243 Egaleo, Greece; klyker@uniwa.gr; 2 2nd Department of Obstetrics and Gynecology, Aretaieio University Hospital, 46 Vasilissis Sofias Avenue, 11528 Athens, Greece; karampasgr@yahoo.gr; 3 Department of Obstetrics, Iasis Private Hospital Paphos, 8 Voriou Ipirou Street, 8036 Paphos, Cyprus; rioulla1@hotmail.com; 4 Neonatal Department, Aretaieio University Hospital, 46 Vasilissis Sofias Avenue, 11528 Athens, Greece; niakobid@med.uoa.gr; 5 Unit of Developmental and Behavioral Pediatrics, 1st Department of Pediatrics, National and Kapodistrian University of Athens, 5 Mikras Asias Street, 11527 Athens, Greece; ppervanid@med.uoa.gr; 6 Unit of Endocrinology, Diabetes Mellitus and Metabolism, Aretaieio University Hospital, 46 Vasilissis Sofias Avenue, 11528 Athens, Greece; gmastorak@med.uoa.gr; 7 Hormone Laboratory, Aretaieio University Hospital, 46 Vasilissis Sofias Avenue, 11528 Athens, Greece; drizos@aretaieio.uoa.gr

**Keywords:** serum, activin A, biomarker, neonatal brain injury, periventricular leukomalacia, intraventricular haemorrhage, premature neonates

## Abstract

Disruption of normal intrauterine brain development is a significant consequence of premature birth and may lead to serious complications, such as neonatal brain injury (NBI). This prospective case-control longitudinal study aimed at determining the levels and prognostic value of serum activin A during the first three days of life in human premature neonates which later developed NBI. It was conducted in a single tertiary hospital and eligible participants were live-born premature (<34 weeks) neonates. Each case (*n =* 29) developed NBI in the form of an intraventricular haemorrhage, or periventricular leukomalacia, and was matched according to birth weight and gestational age to one neonate with normal head ultrasound scans. Serum activin A levels in both groups showed a stable concentration during the first three days of life as no difference was observed within the two groups from the first to the third day. Neonates diagnosed with NBI had significantly higher activin A levels during the first two days of life compared to control neonates and its levels correlated to the severity of NBI during the second and third day of life. Although serum activin A on the second day was the best predictor for neonates at risk to develop NBI, the overall predictive value was marginally fair (area under the ROC-curve 69.2%). Activin A, in combination with other biomarkers, may provide the first clinically useful panel for the early detection of premature neonates at high risk of NBI.

## 1. Introduction

Disruption of normal intrauterine brain development is a significant consequence of premature birth and may lead to serious complications [1]. Several factors play an important role on the odds of neuronal injury, but gestational age (GA) is the main determinant of specific vulnerability to different types of lesions [1,2].

Common lesions of neonatal brain in neonates of 24–32 weeks gestational age and in very low birth weight (<1500 g) neonates are periventricular leukomalacia (PVL) and intraventricular hemorrhage (IVH) [1], while hypoxic-ischemic encephalopathy (HIE) occurs more frequently in late preterm neonates [2]. Despite the advances in perinatal medicine and clinical management of premature neonates (PNs), the prevalence of PVL and IVH remains high, and is estimated at 9.8–34.1% and 10–20%, respectively [1,3]. Neonatal morbidity and adverse neurodevelopmental outcomes do not show decreasing trends, underlining the need for early and individualized therapeutic intervention to prevent severe neonatal brain injury (NBI) [4].

Regardless of ongoing research, for the time being, there is no available effective prognostic model for clinical use, which might provide early detection of neonates at high risk of developing NBI [5]. Ideally, healthcare professionals in Neonatal Intensive Care Units (NICUs) worldwide should have access in a panel of biomarkers for brain injury, so as to be able to identify NBI at early stages, where routine neuroimaging means are still not able to diagnose it [6,7]. A recent review by Bersani et al. provides an up-to-date account of the most promising biomarkers for diagnostic and prognostic use in the management of NBI of varying severity [8], and activin A seems to be one of them [9,10].

Human activin A is a 26 kDa disulfide linked homodimer of two beta A chains, each containing 116 amino acid residues [11]. It belongs to the transforming growth factor beta-family (TGF-β) and exhibits a wide range of biological activities including regulation of cellular proliferation and differentiation, and promotion of neuronal survival [12,13,14,15]. Hypoxic/ischemic, hemorrhagic or infectious insults can cause a strong up-regulation of activin A, since its induction occurs early after brain injury, and it has been proposed that its use may provide a potential biochemical index of the presence, location and extent of brain injury [9,10].

Currently, studies in PNs using activin A as biomarker in different biological fluids, such as amniotic fluid, serum, plasma and urine [10,16,17,18,19,20], have demonstrated adequate efficacy in predicting adverse neonatal outcomes or NBI. However, few of them have enrolled strictly only PNs in their study sample. More specifically, in their cutting edge paper of 2013, Sannia et al. longitudinally measured urine activin A in PNs in the first 72 h after birth and reported that in neonates who developed IVH it was significantly higher than in the controls at all monitoring time-points [10]. Moreover, Shahid et al. [16] investigated the predictive value of amniotic fluid, umbilical cord blood and 3rd day peripheral blood activin A in brain injury (PVL and IVH) in PNs. Their results demonstrated that activin A levels were significantly higher in all the aforementioned biological fluids in the case group when compared to the control group, suggesting that activin A is a useful biomarker in the early prediction of NBI. Additionally, Florio et al. [18,19] evaluated the use of plasma activin A concentrations obtained from umbilical vessels after cord clamping. In the first study, they found that activin A concentrations at birth were increased in PNs who later developed IVH and in the second study they revealed that hypoxic PNs at birth had increased activin A levels in comparison to non-hypoxic PNs. Lastly, Lu et al. [21] evaluated amniotic and cord blood levels of multiple biomarkers among which activin A was the best predictor of long-term brain injury. However, there is still no consensus regarding which biological fluid or method (one sample or multiple longitudinal sampling) is the most effective for the early detection of PNs at a high risk of NBI when activin A is used as a predictor.

We aimed at investigating whether serum activin A levels during the first 3 days of life in PNs (<34 weeks) (a) show a significant variance in time in neonates with (case group) or without (control group) NBI, (b) differ significantly between the control and the study group, and (c) have a predictive value regarding the early identification of PNs at a high risk of developing brain injury.

## 2. Materials and Methods

### 2.1. Study Design

The present study is part of a wider research protocol on the levels and predictive values of NBI biomarkers in PNs [22,23]. The methods of the research study have been previously published [22] and are summarized here as follows:

This is a prospective longitudinal case-control study of live born premature (<34 weeks) neonates, born at a single tertiary private maternity hospital and admitted to the NICU between November 2016–March 2018. Inclusion criteria were (a) written informed consent from neonate’s parents, (b) prematurity < 34 weeks, and (c) NBI in the form of either PVL or IVH for the case group. Neonates with a) major congenital, genetic or chromosomal abnormalities, (b) other types of NBI, such as HIE, (c) congenital brain abnormality based on prenatal and postnatal ultrasounds, (d) congenital TORCH infections, (e) kernicterus, (f) congenital hypothyroidism or untreated maternal thyroid disease, (g) incomplete maternal antenatal care during all trimesters of pregnancy, or (h) maternal use of illicit/addictive substances during pregnancy, were excluded from the specific study.

All neonates were admitted at NICU right after delivery. As per the NICU’s protocols, on admission of every neonate with GA < 34 weeks, blood culture, complete blood count (CBC), C-reactive protein (CRP) and arterial blood gas should be obtained. Routinely, CBC and CRP should be also assessed on the second and third day of life. Blood was collected from peripheral or umbilical vessels. After completion of the standard clinical laboratory tests, any remaining unused amount of serum was used afterwards for the measurement of activin A. The residual serum was stored in aliquots at −35 °C until assayed.

Neonatal brain injury was classified at discharge, taking into account all head ultrasound scans (HUS) during hospitalization. Following on, the neonates were allocated in the case or control group. All HUSs were performed at bedside and evaluated by the Chief Paediatric Radiologist of the Hospital.

### 2.2. Clinical Assessment and Laboratory Data

Medical records were assessed by the research team and any perinatal factors that could be of interest to either influence or predict NBI were recorded. Accuracy of data collection was double checked by the researchers before statistical analysis. Anonymization/deidentification of all participants (mothers and neonates) was preserved through the creation of automatic coding by the database used.

Determination of activin A concentrations was performed with Activin A—AL 110 ELISA kit from Ansh Labs, Webster, TX, USA. According to the manufacturer’s data, the lowest detection limit was 0.065 ng/mL and the precision, as estimated by the total CV (%), was <5.7%. Values < 0.065 ng/mL were reported as zero.

### 2.3. Definitions and Classifications

We defined high-risk pregnancy in accordance with international standards and guidelines including preeclampsia [24], oligohydramnios [25], hypothyroidism [26], gestational diabetes mellitus [27], chorioamnionitis [28], fetal growth restriction [29] and pathological Doppler [30]. Intraventricular hemorrhage was classified in agreement with Papile et al. [31]; periventricular leukomalacia according to de Vries et al. [32], and HUS patterns according to the European Standards of Care for Newborn Health (ESCNH) [33].

### 2.4. Statistical Analysis

The statistical software: IBM SPSS statistics version 23 (IBM Corporation, Somers, NY, USA), was used for data analysis. Statistical analysis was performed and cross-checked by the study personnel. Sample size calculation for this wider research protocol was based on the levels of S100B, which is considered as the “gold standard” of NBI biomarkers [22]. Comparison of maternal and neonatal characteristics was conducted to ensure the success of the matching and to identify the differences between the two groups. Comparisons of qualitative data were done by Pearson’s chi-square test (X^2^). Distribution of concentration of activin A and all other quantitative parameters were tested for normality using the One-Sample Kolmogorov–Smirnov test. Based on this analysis, parametric Student’s *t*-test was used in comparisons of activin A concentration and the other quantitative parameters between groups. Comparison of activin A levels within the two groups during the first three days of life was done by a one-way analysis of variance (ANOVA) test. A subgroup analysis was also performed among control neonates and neonates with either PVL or IVH, so as to investigate whether activin A levels varied in different forms of NBI. Besides, the 5 neonates from the case group that died were compared to control and the rest of the cases, in order to elucidate if activin A is altered in such an adverse neonatal outcome during the first three days of life. Finally, the predictive value of serum activin A regarding NBI was investigated in a multivariate logistic regression model, setting (a) as outcome the presence or not of NBI at discharge from NICU, and (b) as predictive variables the levels of serum activin A. Further analysis on the predictive value of activin A included a second multivariate logistic regression model, which involved the levels of serum activin A and S100B [22] during the first three days of life in the same population of PNs as the predictive variables. A probability level of less or equal to 0.05 was considered significant.

## 3. Results

**Figure 1:**
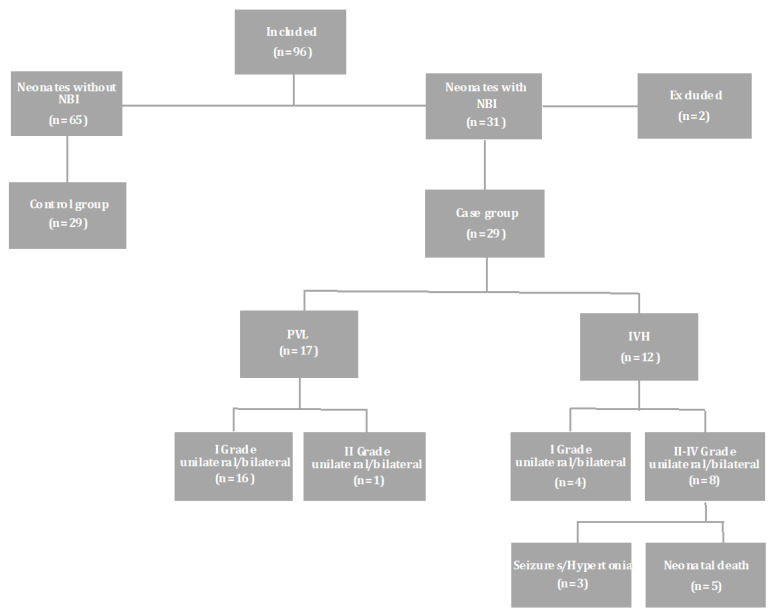
Flowchart of the studied population and perinatal outcome. NBI: neonatal brain injury; IVH: intraventricular haemorrhage; PVL: periventricular leukomalacia. Reprinted from Clinica Chimica Acta, 510, Metallinou D, Karampas G, Nyktari G, Iacovidou N, Lykeridou K, Rizos D, S100B as a biomarker of brain injury in premature neonates. A prospective case—control longitudinal study, 781–786, Copyright (2020), with permission from Elsevier.

Ninety six (*n =* 96) neonates fulfilled the inclusion criteria. Sixty-five (*n =* 65) out of them had normal HUS and comprised the “control group”, while the remaining thirty-one (*n =* 31) developed NBI during hospitalization. From the latter, 2 neonates met the criteria for HIE and were excluded from the study, while the other 29 neonates developed either PVL (*n =* 17) or IVH (*n =* 12) and constituted the “case group”. Sixteen (*n =* 16) neonates in the PVL subgroup developed grade I unilateral or bilateral brain injury and one (*n =* 1) grade II. In the IVH subgroup, 4 neonates developed grade I unilateral or bilateral IVH and the rest 8 neonates grade II–IV unilateral or bilateral IVH. From the latter, 6 neonates presented seizures, 3 of whom died, while the other 3 neonates developed hypertonia in addition to seizures. Overall, 5 neonates in the IVH subgroup died (Figure 1). Finally, neonates from the case group (*n =* 29) were matched in a 1:1 fashion with neonates who had normal HUS (control group) at the same GA within one week, and a similar birth weight.

No differences were observed in maternal characteristics between the two groups, except for the antenatal use of corticosteroids between women whose neonates developed IVH and those that developed PVL (Table 1). On the contrary, significant differences were observed in neonatal demographic-clinical characteristics and laboratory findings between the case and control group. More specifically, admission pH and white blood cell count were significantly lower in the case group, whereas admission base deficit and concentration of lactic acid were higher (Table 2). Furthermore, no differences were observed in therapeutic interventions in neonates between the two groups (Table 3). Finally, regarding neonatal outcomes (Table 4), necrotizing enterocolitis was more frequent in control neonates, while seizures and death were more frequent in the case group.

**Figure 2:**
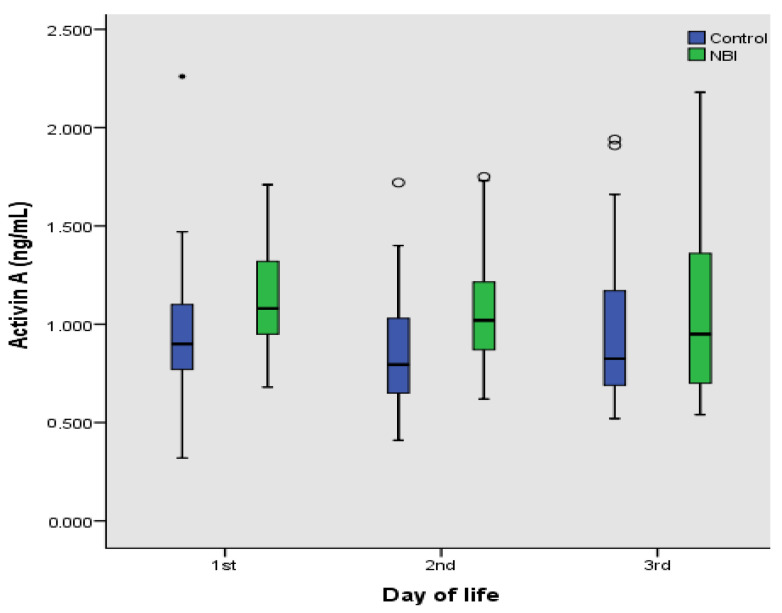
Box plots (horizontal line: median; box: 25–75% percentiles; whiskers: min–max; circles: outliers) of values of Activin A (ng/mL) in control and neonates with neonatal brain injury in (NBI) the first 3 days of life.

Serum activin A levels were available for comparison at 85/87 (97.7%) of the desired time points for both case and control group (Figure 2). Missing data were due to insufficient serum left after routine investigation had been performed. Mean ± Standard Deviation of activin A levels are being presented in Table 5. No differences in activin A levels were observed within each group during the first three days of life. On the contrary, serum activin A was significantly higher in the case group on the first and second day of life (Table 5). Furthermore, Pearson’s rank correlation coefficient (r) revealed that activin A levels did not show any significant correlation with gestational age in control neonates on the 1st (r = 0.089, *p* = 0.666), 2nd (r = 0.124; *p* = 0.546) or 3rd day (r = 0.102; *p* = 0.619) of life.

Further subgroup analysis included a comparison (a) between the 6 neonates in the control group that developed necrotizing enterocolitis and all the other 23 control neonates (b) between the 5 neonates that died from the case group and all other 53 neonates, (c) among control neonates and neonates with either PVL or IVH, and d) between neonates with IVH and those with PVL. Serum activin A in neonates with necrotizing enterocolitis did not differ from that of the other control neonates, while the 5 neonates that died had significantly higher activin A serum concentrations on the 3rd day of life in comparison to all the other neonates (*p =* 0.02). Additionally, neonates with IVH had significantly higher concentrations of serum activin A vs controls on the 1st and 2nd day of life (*p =* 0.043 and *p =* 0.014 respectively). Interestingly, when the 8 neonates from the case group that developed grade II–IV IVH (out of which 5 died) were compared to all other neonates (control plus the rest of the neonates with NBI, *n =* 50), a significant difference was observed on the 2nd and 3rd day of life (*p =* 0.038 and *p =* 0.011 respectively).

**Figure 3:**
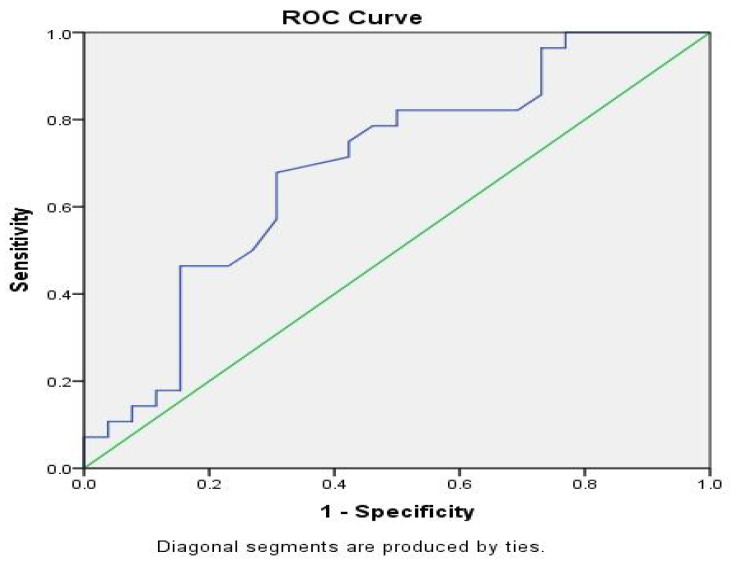
ROC curve for serum activin A on the 2nd day of life between neonates with or without neonatal brain injury. Area under the curve 69.2%, *p =* 0.015, 95%; Confidence Interval: 54.9–83.6%.

The predictive value of serum activin A during the first 3 days of life was evaluated in a multivariate conditional logistic regression model, setting as an outcome the presence or not of NBI at discharge from NICU, and as predictive variables the levels of serum activin A during the first 3 days of life. Using the forward stepwise conditional method, serum activin A on the 2nd day of life was the best predictor for the development of NBI until discharge from the NICU (*p =* 0.022 for the model, *p =* 0.034 and *p =* 0.043 for activin A and the constant respectively). More specifically, a receiver-operating characteristic curve (ROC-curve) for serum activin A on the 2nd day of life yielded a marginally fair predictive value, with an area under the curve at 69.2% (*p =* 0.015, 95%CI: 54.9–83.6%). According to the ROC-curve analysis for a cut-off value of 0.61 ng/mL, sensitivity was 100% and specificity 33.1%, while for a cut-off value of 1.725 ng/mL, sensitivity was 7.1% and specificity 100% (Figure 3). Additional multivariate logistic regression analysis included a second model, setting the levels of serum activin A and S100B during the first three days of life in the same population of PNs as predictive variables. Serum S100B was the most potent predictive variable for the later development of NBI [22], already from the first day of life.

## 4. Discussion

Numerous sources of evidence are available to support that activin A plays an important neuroprotective role in brain injury, demonstrating multiple biological effects, such as regulation of cellular proliferation/differentiation and promotion of neuronal survival [11,12,13,14,15], as its receptors are widely expressed in brain tissue. Specifically, activin A receptor subtype Acvr2a is involved in the regulation of myelin repair after brain injury, featuring that activin A may serve as a novel therapeutic target for the repair of myelin damage [11]. It also promotes the differentiation of oligodendrocyte precursor cells into οligodendrocytes by activating the extracellular signal-regulated kinase and mitogen-activated protein kinase signaling pathway [12]. Moreover, it seems to have a neuroprotective effect by preventing apoptosis via inhibition of diverse intracellular apoptotic pathways [13], while in excitotoxic brain injury, neuroprotection elicits from the activin’s A anti-inflammatory activity [14]. Finally, in hippocampal neurons cultured in vitro, Manickam et al. [15] reported that activin A increases the length and number of synapses of the dendritic aponeurosis neck, also demonstrating a neurotrophic effect.

The biological function of activin A is mediated by the type I and II receptors and by two activin-binding proteins: follistatin and follistatin-related genes. These elements bind to activin A and, by that means, inhibit its biological effects. Research evidence suggests that the expression of activin A is upregulated as a response to acute and chronic neuronal damage of various sources (hypoxic/ischemic injury, mechanical irritation and chemical damage or a combination of these) [14,34]. After an insult, the blood–brain barrier (BBB) permeability changes and is lead to an impaired function, which could explain the elevation of activin A and other biomarkers of brain injury in the serum/plasma of PNs. However, translational research should increasingly focus on the link between BBB dysfunction and brain insults in PNs [35].

Our study is in line with previous studies [16,17,18,36], which demonstrated that activin A levels in PNs, which will develop later on NBI, differ significantly during the first days of life when compared to neonates without NBI. Furthermore, similar to prior studies, we found a correlation between activin A levels and the severity of NBI; neonates in our study that had grade II-IV IVH or died due to severe IVH had significantly higher levels of serum activin A when compared to neonates without or with grade I NBI [16,36]. Interestingly, considering our results on S100B [22], in the same study population and during the same period of neonatal life, serum activin A seems to demonstrate a slow response to NBI, as it rises on either the 2nd or the 3rd day of life in PNs with severe NBI. On the contrary, S100B is already significantly elevated from the 1st day of life, particularly at admission to NICU. To the best of our knowledge, this study is the first one to directly compare activin A levels in PNs with those of S100B, which is considered to be the “gold standard” of NBI biomarkers in PNs [22]. In the study by Shahid et al. [16], a comparison between the ΝΒΙ groups showed that there is a strong correlation between high activin A levels and severe ΝΒΙ, not only on the 3rd day of life but in venous cord blood and amniotic fluid as well. Similar results were reported by Abdel Wahed et al. [36], where a positive correlation between serum activin A concentration and grading of IVH was observed.

Nevertheless, it is worth mentioning that there is a great heterogeneity between similar studies and ours regarding the studied population, focusing mainly on the type and/or on the grade of NBI and the type of biological fluid used in each study. The majority of previous studies refer to premature neonatal population with IVH [17,18,36], while only one study refers to neonates with PVL [16]. However, only the present study provides results on subgroup analysis comparing the PVL or the IVH group with the control group separately. Regarding the biological fluids used in similar studies, apart from peripheral blood, activin A levels have also been evaluated in umbilical artery/vein plasma or serum [16,17,18,19,20,36], amniotic fluid [16] and urine [10]. In all the aforementioned studies, activin A was detected in higher concentrations in PNs with NBI. Consequently, the optimum biological fluid for the assessment of activin A levels and its predictive value in PNs is still debatable.

As currently there is no effective predictive model or technique to detect PNs at high risk of developing brain injury [37], biomarkers of NBI are under investigation for their predictive role. Unfortunately, neuroimaging techniques, such as HUS or MRI, have a limited predictive value in the first hours or days of life [38,39,40]. Thus, brain injury biomarkers may provide the early diagnostic tool for the detection of neonates at a high risk of developing NBI.

Our study, although it is not the first one to evaluate the levels and predictive value of activin A in PNs that will develop NBI, nevertheless has a number of strengths. It is the first study where the levels of serum activin A in the study population are evaluated longitudinally during the first three days of life. Therefore, we originally demonstrate the variance of activin A within groups throughout time, showing that activin A is relatively stable during this period of life in both groups. Moreover, our study is the second one, after that by Shahid et al. [16], which includes not only IVH cases but PVL ones as well, and is the first to provide a subgroup analysis based on NBI subtype. In our study, IVH cases had a higher grade of NBI in comparison to those with PVL. This difference in severity of NBI was represented in the levels of activin A, as neonates with IVH had higher levels when compared to control ones. Finally, an additional strong point of our work lies in the direct comparison of the levels and predictive value of activin A in PNs at high risk of developing NBI to the levels and predictive value of S100B. This fact adds substantially to the growing body of literature with reference to strengths and weaknesses of activin’s A role.

On the contrary, a limitation of our study is the relatively small number of neonates complicated with a severe adverse neonatal outcome. Nevertheless, activin A seems to respond to severe NBI, especially after the first day of life. In combination with other brain injury biomarkers, it could provide an effective predictive model for neonates at risk in the future. Additionally, the severity of NBI is different in the two studied subgroups, as most neonates in the PVL group presented a low grade brain injury, while in the IVH group, they presented a moderate to high grade, explaining why the IVH group presented significantly higher levels of activin A when compared to the control group.

## 5. Conclusions

Currently, the detection of PNs at high risk of developing NBI is not feasible in clinical practice. The ability to predict severe forms of ΝΒΙ in PNs shortly after birth will provide clinicians with the potential to intervene promptly. Serum activin A during the first days of life seems to be a promising biomarker for the prediction of high risk neonates which will later on develop severe NBI. Further research on the predictive value of activin A regarding NBI in PNs is of great interest. Furthermore, in combination with other biomarkers, it may provide the first clinically useful panel for the early detection of neonates at a high risk of NBI.

## Figures and Tables

**Table 1:** Maternal demographic and clinical characteristics of control and cases neonates [22].

Variable *N*	Control 29	Cases 29	*p*-Value
Maternal age (years)	38.4 ± 5.8	37.9 ± 5.7	0.764
Gestational age (weeks)	29.8 ± 2.5	29.6 ± 3.0	0.817
Parity, *n* (%)			
Nullipara	22 (76)	22 (76)	>0.99
Multipara	7 (24)	7 (24)	
Race, *n* (%)			
Caucasian	29 (100%)	29 (100%)	>0.99
Conception, *n* (%)			
Normal	9 (31)	11(38)	
IVF ^1^	20 (69)	18 (62)	0.581
Gestation, *n* (%)			
Single	13 (45)	8 (28)	
Multiple	16 (55)	21 (72)	0.172
Caesarean delivery, *n* (%)	28 (97)	25 (86%)	0.160
Preterm premature rupture of membranes, *n* (%)	9 (31)	9 (31)	>0.99
Preeclampsia, *n* (%)	4 (14)	1 (3.5)	0.160
Oligohydramnios, *n* (%)	2 (7)	2 (7)	>0.99
Hypothyroidism, *n* (%)	15 (52)	11 (38)	0.291
Insulin dependent gestational diabetes mellitus, *n* (%)	4 (14)	2 (7)	0.389
Abnormal Doppler, *n* (%)	3 (10)	6 (20)	0.277
Antenatal steroid administration, *n* (%)	25 (86)	23 (79)	0.487
Neonates with PVL ^2^		16/17	0.019 *
Neonates with IVH ^3^		7/12	
Antenatal magnesium sulphate administration, *n* (%)	11 (38)	15 (52)	0.291
Abruptio placenta, *n* (%)	1 (3.5)	1 (3.5)	>0.99
Clinical chorioamnionitis, *n* (%)	7 (24)	3 (10)	0.164

^1^ IVF: in vitro fertilization; ^2^ PVL: periventricular leukomalacia; ^3^ IVH: intraventricular hemorrhage. * significant values.

**Table 2:** Neonatal demographic—clinical characteristics and laboratory findings of neonates in the control and case group [22].

Variable *N*	Control Group 29	Case Group 29	*p*-Value
Sex, *n* (%)			
Male	13 (45)	12 (41)	
Female	16 (55)	17 (59)	0.791
Apgar score, median (IQR) ^1^			
1st min	8 (2)	8 (3)	0.089
5th min	9 (1)	9 (1)	0.665
Apgar score < 7, *n* (%)			
1st min	5 (17)	9 (31)	0.220
5th min	0 (0)	1 (3.5)	0.274
Birthweight (gr)	1302 ± 429	1225 ± 475	0.517
Body weight on discharge (gr)	2837 ± 576	2613 ± 385	0.134
Head circumference at birth (cm)	27.5 ± 2.5	27.0 ± 3.2	0.533
Head circumference on discharge (cm)	33.5 ± 1.4	33.5 ± 1.1	0.915
Admission, arterial blood			
pH	7.37 ± 0.01	7.29 ± 0.16	0.033 *
Base deficit, mmol/L	4.29 ± 1.92	7.22 ± 6.59	0.037 *
pCO_2_, mmHg	38 ± 10	40 ± 10	0.371
HCO_3_, mmol/L	20 ± 2	19 ± 5	0.087
Lactic acid, mmol/L	3.1 ± 1.2	4.8 ± 4.3	0.05 *
Hb, g/dL	16.5 ± 3.0	16.9 ± 2.0	0.438
WBC ^2^ count, K/µL	11456 ± 9550	9153 ± 5562	0.05 *
CRP ^3^, mg/L	1.0 ± 0.2	1.3 ± 1.5	0.998
Positive CRP at admission, *n* (%)	14 (48)	12 (41)	0.597
CRP 2nd day, mg/L	6.1 ± 8.7	4.9 ± 8.7	0.294
CRP 3rd day, mg/L	7.6 ± 11.1	6.0 ± 7.5	0.809

^1^ IQR: interquartile range; ^2^ WBC: white blood cells; ^3^ CRP: C-reactive protein. * significant values.

**Table 3:** Therapeutic interventions in neonates of control and case group [22].

Variable *N*	Control Group 29	Case Group 29	*p*-Value
Surfacant administration *n* (%)	21 (72)	22 (76)	0.764
Inotrops, *n* (%)	14 (48)	20 (69)	0.145
Caffeine, *n* (%)	26 (90)	25 (86)	0.687
Cardiopulmonary resuscitation, *n* (%)	3 (10)	6 (20)	0.277
Patent ductus arteriosus treatment, *n* (%)	8 (28)	8 (28)	>0.99
	2	0	
Paracetamol	5	7	
Ibuprofen	1	1	
Surgery			
High frequency ventilation, *n* (%)	4 (14)	7 (24)	0.315
Transfusion, *n* (%)	3 (10)	7 (24)	0.164

**Table 4:** Neonatal outcomes of control and case group neonates [22].

Outcome *N*	Control Group 29	Case Group 29	*p*-Value
Seizures, *n* (%)	0 (0)	6 (20)	0.01 *
Hypertonia, *n* (%)	0 (0)	2 (7)	0.15
Death, *n* (%)	0 (0)	5 (17)	0.019 *
Neonates with PVL ^1^		0/17	
Neonates with IVH ^2^		5/12	0.015 *
Positive blood culture, *n* (%)	1 (3.5)	2 (7)	0.553
Positive cerebrospinal fluid culture, *n* (%)	0 (0)	1 (3.5)	0.274
Clinical neonatal sepsis, *n* (%)	1 (3.5)	2 (7)	0.553
Respiratory distress syndrome, *n* (%)	21 (72)	23 (79)	0.539
Chronic lung disease, *n* (%)	6/27 (22)	5/21 (24)	0.897
Necrotizing enterocolitis, *n* (%)	6 (20)	1 (3.5)	0.044 *
Otoacoustic Emissions Test negative, *n* (%)	7/23 (30)	9/21 (43)	0.392
Automated auditory brainstem response test negative, *n* (%)	4/11 (36)	4/10 (40)	0.864
Length of stay in NICU ^3^, days	63 ± 48	45 ± 30	0.089

^1^ PVL: periventricular leukomalacia; ^2^ IVH: intraventricular hemorrhage; ^3^ NICU: neonatal intensive care unit. * significant values.

**Table 5:** Concentrations of Activin-A (ng/mL) in the two studied groups, in the first 3 days of life (Mean ± Standard Deviation (SD)).

	Control	Cases
N	29	29
1st day		
Mean ± SD	0.914 ± 0.361	1.109 ± 0.287 *
2nd day		
Mean ± SD	0.873 ± 0.311	1.06 ± 0.291 **
3rd day		
Mean ± SD	0.987 ± 0.403	1.057 ± 0.436

* *p* = 0.029 compared to control. ** *p* = 0.027 compared to control.

## Data Availability

Not applicable.
